# Notes on correctness of *p*-values when analyzing experiments using SAS and R

**DOI:** 10.1371/journal.pone.0295066

**Published:** 2023-11-30

**Authors:** Razaw Al-Sarraj, Johannes Forkman

**Affiliations:** 1 Department of Energy and Technology, Swedish University of Agricultural Sciences, Uppsala, Sweden; 2 Department of Crop Production Ecology, Swedish University of Agricultural Sciences, Uppsala, Sweden; Medical University of Vienna, AUSTRIA

## Abstract

It is commonly believed that if a two-way analysis of variance (ANOVA) is carried out in R, then reported *p*-values are correct. This article shows that this is not always the case. Results can vary from non-significant to highly significant, depending on the choice of options. The user must know exactly which options result in correct *p*-values, and which options do not. Furthermore, it is commonly supposed that analyses in SAS and R of simple balanced experiments using mixed-effects models result in correct *p*-values. However, the simulation study of the current article indicates that frequency of Type I error deviates from the nominal value. The objective of this article is to compare SAS and R with respect to correctness of results when analyzing small experiments. It is concluded that modern functions and procedures for analysis of mixed-effects models are sometimes not as reliable as traditional ANOVA based on simple computations of sums of squares.

## Introduction

In this digital age, researchers are presented with an unprecedented opportunity to analyze a wide range of data quickly and efficiently using statistical software. While these software aim to employ the same methods for data analysis, the results they produce can vary broadly, which in turn leads to different conclusions being drawn. Sometimes, conclusions may even be incorrect. Hence comes the importance of the statistical method for data analysis.

Within the various software, an array of options are available to analyze linear fixed-effects and mixed-effects models. Here, the question posed is as to which of the results obtained are more accurate. We examine functions in R [1] and procedures of SAS [2] for linear fixed-effects and mixed-effects models. For the fixed-effects case, we discuss unbalanced two-factorial experiments and type 3 tests, while for the mixed-effects case, we examine balanced randomized complete block experiments and split-plot experiments with focus on Type I error.

In fixed-effects models, there are exact F-tests, calculated from sums of squares in classical analysis of variance table [3]. There are different types of sums of squares commonly known as types 1, 2 and 3. For balanced datasets, results are identical, whereas for unbalanced datasets the different types are associated with different null hypotheses [4]. There have been and still are many different viewpoints regarding the types of sums of squares [5, 6]. The following are recurring arguments: Type 1 sums of squares are useful when cell frequencies are good estimates of population proportions [7]; Type 2 sums of squares have better power than type 3 sums of squares when interaction is negligible [8]; and type 3 sums of squares are appropriate when observations are missing at random, as their null hypotheses are not dependent on the cell frequencies [4, 9]. The choice of type depends on the question the researcher wants to answer [10].

Nowadays, the linear mixed-effects model is used extensively and has in practice overrun the classical analysis of variance [11]. The linear mixed-effects models are advantageous when applied in an array of sophisticated problems. The F-tests obtained using linear mixed-effects models are, however, in general approximate. The F-test statistics are no longer ratios of simple mean squared errors from analysis of variance, but are computed using linear functions of parameter estimates. These statistics are also functions of variance components associated with random effects. Variance components can be estimated by equating the observed mean squares to their expected values in a simple analysis of variance setting, or by using likelihood-based methods such as the restricted maximum likelihood (REML) method [12, 13]. Although the computed variance components can be negative [11, 14], they can still be interpreted in a legitimate manner [15, 16]. In practice, the estimates of the variance components are constrained to be non-negative, which may affect the assumption on the appropriate number of degrees of freedom in the denominator of the F-distribution. Different methods for computing the denominator degrees of freedom are available [17]. The Kenward and Roger method [18, 19] is recommended [20–23]. However, this method is implemented differently in SAS and R with regard to how the number of degrees of freedom is calculated when estimates of the variance components are zero. This poses a problem at hypothesis testing of fixed effects, because different software give different results.

Bearing in mind the many limitations associated with traditional analysis of variance, the linear mixed-effects model has gained a broader application. It is however necessary to carry out the analysis carefully, even in simple cases. In this study, we investigate the implications of the available options in the different software with regard to tests of fixed effects in linear fixed-effects and mixed-effects models.

As regards fixed-effects models, we are particularly interested in the type 3 tests provided by the Anova function of the car package [24] and the ezANOVA function of the ez package [25] in R. It has been noted that the Anova function ‘does not always give the same results as SAS does.’ [5] From a practical point of view, this important observation deserves more attention. We will present an example that displays differences in *p*-values computed in SAS and R, depending on the procedure or function used. Through this example, we shall clarify what null hypotheses are actually tested.

Regarding mixed-effects models, we investigate how R and SAS handle non-positive estimates of variance components and what consequences it has for the *p*-values. In our experience, non-positive estimates are common. We will draw attention to the fact that statistical packages handle this situation differently and discuss the alternatives.

The next section, Background and methods, recalls the theory and specify models. The Examples section provides illustrating examples. The unbalanced two-factorial experiment uses a fixed-effects model and notes differences between R and SAS with regard to results of type 3 tests. The randomized complete block and split-plot experiments use mixed-effects models with non-positive estimates of variance components and observes differences between R and SAS regardless of the type of test. The Simulation studies section investigates mixed-effects models further. In this section, frequency of Type I error is estimated through simulation. The Practical advice section gives useful recommendations. The article ends with a Discussion.

## Background and methods

### General model

A general fixed-effects linear model can be written as

$$\begin{array}{c} Y=X\beta +e, \end{array}$$ (1)

where *Y* is an *n* × 1 vector of observations, *X* is a known *n* × *m* design matrix, *β* is an *m* × 1 vector of unknown fixed-effects parameters, *e* is an *n* × 1 vector of normally distributed random effects assumed to have mean 0 and variance-covariance matrix $\sigma_{e}^{2}I_{n}$, where *I*_*n*_ is an *n* × *n* identity matrix. If *X* is of full rank, then the best linear unbiased estimator is obtained using the least-squares solution $\hat{\beta }={\left(X^{\prime }X\right)}^{-1}X^{\prime }Y$. Otherwise, if *X* is not of full rank, i.e., if some columns are linearly dependent, the model is said to be overparameterized and the inverse of *X*′*X* cannot be computed. Instead a generalized inverse, (*X*′*X*)^−^, can be used, and the solution will be $\hat{\beta }={\left(X^{\prime }X\right)}^{-}X^{\prime }Y$ [3]. This solution is not unique, because it depends on the choice of the generalized inverse. In practice, the sweep operator may be used for the computation [26]. Using this method, the least-squares solution depends on the ordering of the columns of the design matrix *X*. However, there are some linear functions of the parameters, the so-called estimable functions, that have unique solutions.

A linear hypothesis can in general be expressed as

$$\begin{array}{c} H_{0}:L\beta =0, \end{array}$$ (2)

where *L* is a vector or matrix that is a linear combination of the rows of *X*, which makes *Lβ* estimable even if the model is overparametrized. This hypothesis (2) can be tested using the *F*-statistic

$$\begin{array}{c} F=\frac{Q_{A}/k}{{{\hat{\sigma }}_{e}}^{2}}, \end{array}$$ (3)

where $Q_{A}={\left(L\hat{\beta }\right)}^{\prime }{\left(L{\left(X^{\prime }X\right)}^{-}L^{\prime }\right)}^{-1}\left(L\hat{\beta }\right)$, *k* = rank(*L*), and ${\hat{\sigma }}_{e}^{2}$ is the estimated error variance, i.e., the mean square error. Under the null hypothesis, *F* is F-distributed with *k* and *n* − rank(*X*) degrees of freedom.

Another way to deal with the problem of an overparametrized model is through putting constraints on the parameters. Usually this is done by setting the parameters corresponding to the first or last levels of the factors to zero, or just constrain the parameters of each factor in such a way that they sum up to zero, which is called sigma-restriction [4].

Adding random effects to model (1) gives the so-called mixed-effects model:

$$\begin{array}{c} Y=X\beta +Zu+e, \end{array}$$ (4)

where *Z* is an *n* × *q* incidence matrix of known elements, and *u* is a *q* × 1 vector of unknown random effects. It is assumed that *u* and *e* are independently distributed, and *u* ∼ *N*(0, *G*), where *G* is a block-diagonal variance-covariance matrix. Hence, *Y* ∼ *N*(*Xβ*, *V*), where $V=ZGZ^{\prime }+\sigma_{e}^{2}I_{n}$. When estimates are substituted for the variance components of *V*, this matrix is denoted $\hat{V}$. The hypotheses (2) can be tested using the test statistic

$$\begin{array}{c} F=\frac{{\left(L\hat{\beta }\right)}^{\prime }{\left(L\hat{C}L^{\prime }\right)}^{-1}\left(L\hat{\beta }\right)}{k}, \end{array}$$ (5)

where $\hat{\beta }={\left(X^{\prime }{\hat{V}}^{-1}X\right)}^{-}X^{\prime }{\hat{V}}^{-1}Y$ is the estimate of the fixed effects, *β*, and $\hat{C}={\left(X^{\prime }{\hat{V}}^{-1}X\right)}^{-}$ is the estimated variance-covariance matrix of $\hat{\beta }$. This *F*-statistic is approximately F-distributed with $k=\text{rank}(L\hat{C}L^{\prime })$ and $\hat{\upsilon }$ degrees of freedom, where $\hat{\upsilon }$ must be estimated. In the software we consider, there are a number of available methods for computing *υ*, among them the Kenward and Roger method [19, 20].

### A two-way fixed-effects model for unbalanced data

In the context of analysis of variance, we consider a special case of model (1), a two-way fixed-effects model with interaction:

$$\begin{array}{c} y_{ijk}=\mu +\alpha_{i}+\beta_{j}+\gamma_{ij}+e_{ijk};\;i=1,\ldots ,a;\;j=1,\ldots ,b,\;k=1,\ldots ,n_{ij}, \end{array}$$ (6)

where *μ* is an intercept, and *α*_*i*_ and *β*_*j*_ are unknown fixed effects for the factors *A* and *B*, respectively. The interaction of the *i*-th and *j*-th level of the two fixed factors *A* and *B* is represented by *γ*_*ij*_. The experimental errors, *e*_*ijk*_, are assumed independently distributed, $e_{ijk}∼N\left(0,\sigma_{e}^{2}\right)$.

The data can be written in a table with *a* rows, *b* columns and *n*_*ij*_ observations in the *ij*-th cell. Notice that this two-way table has no empty cells, i.e., *n*_*ij*_ ≥ 1, but the numbers of observations may vary between the cells. The total number of observations is $n_{..}=\sum_{i=1}^{a}\sum_{j=1}^{b}n_{ij}$, and the cell means are ${\bar{y}}_{ij.}=\sum_{k=1}^{n_{ij}}y_{ijk}/n_{ij}$. Furthermore, let *μ*_*ij*_ denote the expected value in *ij*-th cell, i.e., *μ*_*ij*_ = *μ* + *α*_*i*_ + *β*_*j*_ + *γ*_*ij*_.

There are three commonly used computing procedures with regard to sums of squares and tests, known as types 1,2 and 3. When data is balanced they are all equal; however, in unbalanced data they are usually not. The type 1 test is a forward sequential procedure. First, the sum of squares for one factor, say *A*, is computed without the other factor, *B*, in the model. Next, the sum of squares for *B* is computed, given that *A* is already in the model. With this type of test, the result depends on which of the two factors is specified first. For types 2 and 3, the order does not matter. Using type 2, the sum of squares for *A* is computed given that factor *B* is already in the model, whereas the sum of squares for *B* is computed given that *A* is already in the model. The type 3 test computes sums of squares for *A* that are adjusted for effects of *B* and orthogonal to effects of *A* × *B* interaction [6]. Similarly, the sums of squares for *B* are adjusted for *A* and orthogonal to *A* × *B*. The type 3 test was defined by SAS [2, 6] and is the one that tests the null hypothesis of no differences between the unweighted marginal means.

Specifically, the three types of sums of squares test the following null hypotheses [9, 27]. Provided that *A* is specified before *B* at the analysis, the type 1 test of *A* tests the null hypothesis

$$\begin{array}{c} H_{0}:\frac{1}{n_{1.}}\sum_{j=1}^{b}n_{1j}\;\mu_{1j}=\frac{1}{n_{2.}}\sum_{j=1}^{b}n_{2j}\;\mu_{2j}=\cdots =\frac{1}{n_{a.}}\sum_{j=1}^{b}n_{aj}\;\mu_{aj}\;, \end{array}$$ (7)

where *n*_*i*._ is the total number of observations for the *i*-th level of factor A. Each cell mean *μ*_*ij*_ is weighted with respect to the number of observations in the *ij*-th cell. The type 2 test of *A* tests the null hypothesis

$$\begin{array}{c} H_{0}:\sum_{j=1}^{b}n_{ij}\;\mu_{ij}=\sum_{j=1}^{b}\sum_{k=1}^{a}\frac{n_{ij}\;n_{kj}\;\mu_{kj}}{n_{.j}}\;\;,\;\text{for}\;\;i=1,2,\ldots ,a\;, \end{array}$$ (8)

where *n*_.*j*_ is the total number of observations for the *j*-th level of factor B. The type 3 test of *A* tests the null hypothesis

$$\begin{array}{c} H_{0}:\mu_{1.}=\mu_{2.}=\cdots =\mu_{a.}\;, \end{array}$$ (9)

where $\mu_{i}.=\sum_{j=1}^{b}\mu_{ij}/b$ is the unweighted marginal mean of the *i*-th treatment, i.e., all the cells are weighted equally, regardless of their cell frequencies.

The three types of tests can be carried out through computations on reductions of sums of squares [4]. For the type 3 test, this method requires sigma-restricted parametrization. However, the type 3 test coincides with Yates’s method of weighted squares of means [28], which does not require sigma-restriction [4, 6]. Commonly, software neither uses the reduction-of-sums-of-squares method nor the method of weighted squares of means for computations of type 1–3 tests. Instead the general test statistic (3) is used, where *L* may be specified, depending on the type of test, using the Forward-Doolittle operator [26].

### Three mixed-effects models for balanced data

We consider three balanced mixed-effects models (4); the first is a model for analysis of randomized complete block experiments with random effects of blocks:

$$\begin{array}{c} y_{ij}=\mu +\alpha_{i}+b_{j}+e_{ij},\;i=1,\ldots ,a;\;j=1,\ldots ,b\;, \end{array}$$ (10)

where *y*_*ij*_ is the response of the *i*-th level of the fixed effect *A* and the *j*-th level of the random effect *B*. The intercept is denoted as *μ*, the fixed effect of treatment as *α*_*i*_, the random effect of block as $b_{j}∼N\left(0,\sigma_{B}^{2}\right)$, and the residual error effect as $e_{ij}∼N\left(0,\sigma_{e}^{2}\right)$. Let ${\bar{y}}_{i.}=\sum_{j=1}^{b}y_{ij}/b$, ${\bar{y}}_{.j}=\sum_{i=1}^{a}y_{ij}/a$ and ${\bar{y}}_{..}=\sum_{i=1}^{a}{\bar{y}}_{i.}/a$. The expected values can be expressed as *μ*_*i*_ = *μ* + *α*_*i*_.

Under the assumption of model (10), the null hypothesis of no difference between the fixed treatment effects, *H*_0_ : *μ*_1_ = *μ*_2_ = … = *μ*_*a*_, can be performed using the *F*-statistic

$$\begin{array}{c} F=\frac{x_{A}/\left(a-1\right)}{x_{E}/\left(\left(a-1\right)\left(b-1\right)\right)}, \end{array}$$ (11)

where $x_{A}=b\sum_{i=1}^{a}{\left({\bar{y}}_{i.}-\bar{y_{..}}\right)}^{2}$ and $x_{E}=\sum_{i=1}^{a}\sum_{j=1}^{b}{\left(y_{ij}-{\bar{y}}_{i.}-{\bar{y}}_{.j}+{\bar{y}}_{..}\right)}^{2}$. This test statistic is exactly F-distributed with *a* − 1 and (*a* − 1)(*b* − 1) degrees of freedom. Note that (11) is a function of sums of squares and degrees of freedom only. The test is therefore independent of which method is used to estimate the variance components. In particular, it does not matter whether the estimate of $\sigma_{B}^{2}$ is positive or not.

Next, we consider a split-plot experiment. With random effects of blocks, the following mixed-effects model can be used:

$$\begin{array}{c} y_{ijk}=\mu +\alpha_{i}+b_{j}+{\left(ab\right)}_{ij}+\tau_{k}+{\left(\alpha \tau \right)}_{ik}+e_{ijk},\;i=1,\ldots ,a;\;j=1,\ldots ,b;\;k=1,\ldots ,c\;, \end{array}$$ (12)

where *y*_*ijk*_ is the observation in the *k*-th subplot in the *i*-th main plot of the *j*-th block. In (12), *μ* is the intercept, *α*_*i*_ is the fixed effect of the main-plot-treatment factor, *b*_*j*_ is the random effect of the block factor, *τ*_*k*_ is the fixed effect of the subplot factor, and (*ατ*)_*ik*_ is the effect of interaction. The random effects in the model are assumed independently and normally distributed: $b_{j}∼N\left(0,\sigma_{B}^{2}\right)$, ${\left(ab\right)}_{ij}∼N\left(0,\sigma_{AB}^{2}\right)$, and $e_{ijk}∼N\left(0,\sigma_{e}^{2}\right)$. Let ${\bar{y}}_{ij.}=\sum_{k=1}^{c}y_{ijk}/c$, ${\bar{y}}_{i..}=\sum_{j=1}^{b}\sum_{k=1}^{c}y_{ijk}/\left(bc\right)$, ${\bar{y}}_{.j.}=\sum_{i=1}^{a}\sum_{k=1}^{c}y_{ijk}/\left(ac\right)$ and ${\bar{y}}_{...}=\sum_{i=1}^{a}\sum_{j=1}^{b}\sum_{k=1}^{c}y_{ijk}/\left(abc\right)$. The expected values can be denoted *μ*_*ik*_ = *μ* + *α*_*i*_ + *τ*_*k*_ + (*ατ*)_*ik*_.

With fixed effects of blocks, the model can be written:

$$\begin{array}{c} y_{ijk}=\mu +\alpha_{i}+\beta_{j}+{\left(ab\right)}_{ij}+\tau_{k}+{\left(\alpha \tau \right)}_{ik}+e_{ijk},\;i=1,\ldots ,a;\;j=1,\ldots ,b;\;k=1,\ldots ,c\;, \end{array}$$ (13)

where *β*_*j*_ are fixed block-effects, and all other terms are defined as in model (12).

The null hypotheses of no difference between the effects of the main-plot treatments, *H*_0_ : *μ*_1_. = *μ*_2_. = … = *μ*_*a*._, where $\mu_{i.}=\sum_{k=1}^{c}\mu_{ik}/c$ can be tested in the models (12) and (13) using the exact F-test,

$$\begin{array}{c} F=\frac{x_{A}/\left(a-1\right)}{x_{AB}/\left(\left(a-1\right)\left(b-1\right)\right)}, \end{array}$$ (14)

where $x_{A}=\sum_{i=1}^{a}\sum_{j=1}^{b}\sum_{k=1}^{c}{\left({\bar{y}}_{i..}-{\bar{y}}_{...}\right)}^{2}$ and $x_{AB}=\sum_{i=1}^{a}\sum_{j=1}^{b}\sum_{k=1}^{c}{\left({\bar{y}}_{ij.}-{\bar{y}}_{i..}-{\bar{y}}_{.j.}+{\bar{y}}_{...}\right)}^{2}$ are the sums of squares for main-plot treatments and interaction, respectively. This test statistic is exactly F-distributed with *a* − 1 and (*a* − 1)(*b* − 1) degrees of freedom. Unlike (5), which is used by most software packages for mixed-effects models, (14) does not require any estimates of the variance components. Thus, it does not matter whether the estimates of $\sigma_{B}^{2}$ and $\sigma_{AB}^{2}$ are positive or not.

## Examples

This section includes two subsections. The first of these illustrates differences between the three types of tests in an unbalanced two-factorial experiment. Specifically, the issue of the computation of the type 3 test is discussed. The model is a fixed-effects model with a single error variance. The estimate of this variance is always positive, so the issue of non-positive variance estimates is not investigated. The second subsection illustrates differences between methods for computing F-tests in balanced randomized complete block and split-plot experiments. Mixed-effects models are used. Differences between methods (5), (11) and (14) for computing the F-statistics are investigated in presence of non-positive estimates of variances components.

### Unbalanced two-factorial experiment

Here we present an example using model (6). Weight gain was registered (Table 1) after subjecting each of fifteen animals to one of three different diets (B = Diets). We were specifically interested in the effect of sex (A = Sex), which is specified as the first factor in the model. The data was analyzed using all three types of tests (1–3), and the analysis was carried out using the glm procedure [2, 29] in SAS and the lm, anova and Anova functions [1, 24] in R. The anova function, with a small initial “a”, was used for the type 1 test, whereas the Anova function of the car package [24], with capital “A”, was applied for the types 2 and 3 tests. In addition, the ezANOVA function of the ez package [25] and the aov1, aov2, aov3 and GLM functions of the sasLMpackage [30] were investigated. The R script and the SAS program are provided as supporting information in S1 and S2 Files, respectively.

**Table 1:** Observed weight gain in an unbalanced two-factorial experiment.

	Diet 1	Diet 2	Diet 3
Female	10	19, 21	23, 22, 25
Male	13, 17, 14	18, 20, 17	17, 18, 20

In R, parameters of factors are constrained through coding of factor levels. In this work, we have investigated three options for coding: contr.treatment, which is the default option, contr.sum and contr.SAS. With contr.treatment, the first level of the factor is the reference level, i.e., the parameter corresponding to that level is set to zero, whereas with contr.SAS, the last level is the reference level. With contr.sum, however, there is no reference level, but instead the parameters of the levels are sigma-restricted, i.e., their sum is constrained to zero [24].

Table 2 presents the results of the analyses, when using the lm, anova and Anova functions in R and the glm procedure in SAS. The three types of tests gave very different *F*- and *p*-values. Likewise in R, the results with respect to each contrast were highly dependent on the type of test. Regarding the results of types 1 and 2, all methods gave the same result.

**Table 2:** Results from analyses of the unbalanced two-factorial experiment using the lm, anova and Anova functions of R and the glm procedure of SAS: *F*-statistics and *p*-values for the test of sex.

Method	Type 1		Type 2		Type 3	
	*F*	*p*	*F*	*p*	*F*	*p*
R contr.treatment	10.9622	0.0091	3.8073	0.0828	5.9595	0.0373
R contr.sum	10.9622	0.0091	3.8073	0.0828	0.5151	0.4911
R contr.SAS	10.9622	0.0091	3.8073	0.0828	13.6824	0.0049
R proc glm	10.9622	0.0091	3.8073	0.0828	0.5151	0.4911

Since the dataset is unbalanced, the three types of tests are testing different hypotheses. Consequently, the three types were expected to give different *F*- and *p*-values. However, within each type of tests, there should be no differences between the methods. The observed differences between the methods for type 3 were not expected.

For type 1, the null hypothesis is

$$H_{0}:\frac{1}{6}\;\left(\mu_{11}+2\;\mu_{12}+3\;\mu_{13}\right)=\frac{1}{3}\;\left(\mu_{21}+\mu_{22}+\mu_{23}\right),$$ (15)

and for type 2, the null hypothesis is

$$H_{0}:\frac{3}{4}\;\mu_{11}+\frac{6}{5}\;\mu_{12}+\frac{3}{2}\;\mu_{13}=\frac{3}{4}\;\mu_{21}+\frac{6}{5}\;\mu_{22}+\frac{3}{2}\;\mu_{23}\;.$$ (16)

For type 3, correct results were obtained only using the contr.sum parametrization in R or using SAS. The correct type 3 hypothesis is

$$H_{0}:\frac{1}{3}\;\left(\mu_{11}+\mu_{12}+\mu_{13}\right)=\frac{1}{3}\;\left(\mu_{21}+\mu_{22}+\mu_{23}\right).$$ (17)

The test of sex presented by the Anova function as a type 3 test when using the contr.treatment parametrization is a test of the null hypothesis *H*_0_ : *α*_2_ = 0. With this paramerization, *α*_1_ = *β*_1_ = *γ*_11_ = *γ*_12_ = *γ*_13_ = *γ*_21_ = 0. Because of these constraints, *μ*_11_ = *μ* and *μ*_21_ = *μ* + *α*_2_. Thus, the null hypothesis *H*_0_: *α*_2_ = 0 is equivalent to

$$\begin{array}{c} H_{0}:\mu_{11}=\mu_{21}\;. \end{array}$$ (18)

In other words, this test of the factor sex is a test of no difference between males and females, provided they take the first diet. Specifically, it should be noted that this test is not a type 3 test, i.e., it does not test the null hypothesis (17). Since contr.treatment is the default parametrization and no warning is provided in the R console, it is likely that this conditional test is often mistaken for a type 3 test. Similarly, using the contr.SAS parametrization, the test of sex presented as type 3 does not test the type 3 hypothesis (17), but is a test of no difference between males and females when conditioned on the third diet.

SAS does not put any constraints on the parameters but uses the Forward-Doolittle factorization [26, 31] to determine the *L* that gives the correct type 3 test. It should be noted that both the glm procedure of SAS and the Anova function of the car package in R uses (3) for computation of the *F*-statistic, but these methods, glm and Anova, specify *L* differently.

The ezANOVA function of R gave the same results as those presented for R in Table 2. Thus, the type 3 tests were incorrectly computed when contr.treatment and contr.SAS were used. In contrast, the aov1, aov2, aov3 and GLM functions produced the same results as the glm procedure of SAS. In this example, these functions performed types 1–3 tests correctly.

### Randomized complete block and split-plot experiments

In the following examples, the mixed procedure [2, 15] in SAS and the lmer function [32] in R were used for fitting models with both fixed and random effects, i.e., mixed-effects models (4). With both the mixed procedure and the lmer function, the variance components were estimated using the restricted maximum likelihood (REML) method. Using lmer, variance components are bounded to be non-negative. This is also the default setting of the mixed procedure. However, using the nobound option of the mixed procedure, negative estimates of the variance components are allowed. The analyses were carried out using version 9.4 of SAS and version 4.2.2 of R.

Three examples following the models (10), (12) and (13) were considered. In the randomized complete block model (10), we studied tests of treatments. In the split-plot models (12) and (13), we studied tests of main-plot treatments. Exact F-tests were calculated using (11) for model (10), and (14) for models (12) and (13). In both SAS and R, the *F*-values were computed as in (5), and the Kenward and Roger method [18, 19], as implemented in the software, was used for computation of denominator degrees of freedom. Datasets, SAS programs and R scripts are included as supporting information. Results are summarized in Table 3.

**Table 3:** Results of hypothesis testing in the three examples. Denominator degrees of freedom (df), *F*-values and *p*-values using different methods.

	Example 1			Example 2			Example 3		
	df	*F*	*p*	df	*F*	*p*	df	*F*	*p*
Exact F-test	3	5.54	0.100	2	22.82	0.042	2	11.72	0.079
SAS mixed, nobound	3	5.54	0.100	2	22.82	0.042	2	11.72	0.079
SAS mixed, default	6	8.58	0.026	35	9.58	0.001	35	6.18	0.005
R lmer	3	8.58	0.061	2	9.58	0.094	2	6.18	0.139

In Example 1, a randomized complete block experiment with *a* = 2 treatment levels and *b* = 4 block levels was analyzed using model (10). Variance component estimates were ${\hat{\sigma }}_{B}^{2}=-0.1063$ and ${\hat{\sigma }}_{e}^{2}=0.2996$ using the mixed procedure with the nobound option. Without this option or using lmer, variance component estimates were ${\hat{\sigma }}_{B}^{2}=0$ and ${\hat{\sigma }}_{e}^{2}=0.1932$. Using the exact F-test (11), denominator degrees of freedom were calculated as (*a* − 1)(*b* − 1), which agrees with the results obtained using the nobound option of the mixed procedure. These methods also agree with regard to *F*- and *p*-values. Using the default setting of the mixed procedure, denominator degrees of freedom were calculated as *a*(*b* − 1), i.e., as if using a one-way ANOVA model. This computation gave a significant result (*p* = 0.026), while the computation using the nobound option did not (*p* = 0.100). With lmer, denominator degrees of freedom were the same as obtained with the nobound option of the mixed procedure. However, the *F*-value was the same as obtained with the mixed procedure without using the nobound option. Notice that with default settings, SAS and R gave different results. In the former, the effects of treatments were significant (*p* = 0.026), but in the latter they were not (*p* = 0.061). Neither software package provided the exact F-test value by default.

In Example 2, a split-plot experiment was analyzed using model (12). There were *b* = 2 blocks, *a* = 3 main plots per block and *c* = 12 subplots per main plot. Using the nobound option of the mixed procedure, the estimates of the variance components were ${\hat{\sigma }}_{B}^{2}=15.5005$, ${\hat{\sigma }}_{AB}^{2}=-0.04346$ and ${\hat{\sigma }}_{e}^{2}=0.8775$. Without this option, or using lmer, the estimates were ${\hat{\sigma }}_{B}^{2}=15.487$, ${\hat{\sigma }}_{AB}^{2}=0$ and ${\hat{\sigma }}_{e}^{2}=0.848$. The results of the exact F-test and the mixed procedure were the same when the nobound option was used. The denominator degrees of freedom obtained using lmer were equal or close to the correct number (*a* − 1)(*b* − 1) = 2. While the result using the nobound option of the mixed procedure was significant, (*p* = 0.042), it was highly significant (*p* = 0.001) without this option. Using the default settings of the mixed procedure, denominator degrees of freedom were computed as (*ac* − 1)(*b* − 1) = 35. The same *F*-value, 9.58, was obtained using the mixed procedure with default settings and using lmer. The results using the default settings of SAS and R were very different. In SAS, effects of main-plot treatments were highly significant (*p* = 0.001); however in R they were not (*p* = 0.094).

In Example 3, another dataset, with the same experimental layout as in Example 2, was analyzed, but using model (13). In this example, block effects were considered fixed. Unbounded variance component estimates (proc mixed, nobound) were ${\hat{\sigma }}_{AB}^{2}=-0.0441$ and ${\hat{\sigma }}_{e}^{2}=1.0856$, and bounded (proc mixed, default and lmer) were ${\hat{\sigma }}_{AB}^{2}=0$ and ${\hat{\sigma }}_{e}^{2}=1.055$. With fixed effects of blocks, results using the mixed procedure with the nobound option were the same as with the exact F-test. As in Example 2, the *p*-value differed between the default SAS and R.

The results of Table 3 were all produced using type 3 tests. In R, the contr.sum parametrization was used, and the tests were provided by the lmerTest package [33]. Note that the differences between the methods in Table 3 are not due to type 3 test calculations, because the examples are balanced. The differences are explained by the fact that the exact F-test uses equations (11) and (14), while the other three methods all use equation (5) but with different variance estimates and degrees of freedom.

Neither the type of test nor the choice of parametrization should matter when the dataset is balanced. However, it was noted that with types 1 and 2 tests, the *F*-statistic was occasionally computed as 0 when contr.treatment or contr.SAS was used, whereby the *p*-value was correspondingly computed as 1. In SAS, the same phenomenon occurred when analyzing Example 3 using model (12).

## Simulation studies

### Design

In the randomized complete block and split-plot examples, methods gave different results although datasets were balanced. Since those were just a few examples, a simulation study was performed, exploring a wider range of datasets. Specifically, the Type I error rates of the different options for hypothesis testing were estimated.

For each of the models, six layouts, i.e., combinations of *a*, *b* and *c* were investigated, as specified in the first columns of Tables 4 and 5, for model (10) and (12), respectively. In model (10), the error variance was $\sigma_{e}^{2}=1$, but different values were adopted for the block variance: $\sigma_{B}^{2}=0.1,0.3,0.5,0.7,0.9$. In model (12), the block and split-plot error variances were $\sigma_{b}^{2}=1$ and $\sigma_{e}^{2}=1$, respectively, while the main plot error variance varied: $\sigma_{AB}^{2}=0.1,0.3,0.5,0.7,0.9$. All fixed parameters, i.e., *μ*, *α*_*i*_, *τ*_*k*_ and (*ατ*)_*ik*_ in models (10) and (12) were zero. For each case, i.e., row of Tables 4 and 5, 200 000 datasets were generated, and for each generated dataset, the null hypothesis *H*_0_ : *μ*_1_ = *μ*_2_ = … = *μ*_*a*_ in model (10) and *H*_0_ : *μ*_1._ = *μ*_2._ = … = *μ*_*a*._ in model (12), was tested at significance level 0.05. With 200 000 simulations, an approximate 0.95 tolerance interval of ±1.96(0.05(1 − 0.05)/200000)^1/2^ = ±0.001 was achieved. In addition, 10 000 datasets were generated for each case to estimate the probability of a non-positive estimate of $\sigma_{B}^{2}$ in (10) and $\sigma_{AB}^{2}$ in (12).

**Table 4:** Frequency of Type I error at significance level 0.05 and frequency of a non-positive variance estimate, using the randomized complete block model (10).

*a*	*b*	$\sigma_{\text{B}}^{2}$	R	SAS nobound	SAS default	Exact F-test	Pr(${\hat{\sigma }}_{\text{B}}^{2}\leq 0)$
2	2	0.1	0.052	0.052	0.072	0.050	0.448
2	2	0.3	0.051	0.050	0.067	0.049	0.405
2	2	0.5	0.055	0.050	0.066	0.050	0.377
2	2	0.7	0.051	0.051	0.065	0.050	0.348
2	2	0.9	0.051	0.050	0.064	0.050	0.335
2	4	0.1	0.054	0.050	0.067	0.050	0.426
2	4	0.3	0.052	0.050	0.061	0.050	0.352
2	4	0.5	0.052	0.050	0.059	0.050	0.288
2	4	0.7	0.052	0.050	0.057	0.050	0.236
2	4	0.9	0.052	0.050	0.056	0.050	0.201
3	2	0.1	0.052	0.054	0.068	0.050	0.504
3	2	0.3	0.051	0.053	0.066	0.051	0.435
3	2	0.5	0.051	0.053	0.064	0.051	0.385
3	2	0.7	0.051	0.053	0.062	0.050	0.353
3	2	0.9	0.050	0.052	0.061	0.050	0.332
3	4	0.1	0.054	0.050	0.062	0.051	0.431
3	4	0.3	0.053	0.050	0.057	0.050	0.305
3	4	0.5	0.052	0.050	0.055	0.050	0.230
3	4	0.7	0.051	0.050	0.054	0.050	0.181
3	4	0.9	0.051	0.050	0.053	0.050	0.144
4	4	0.1	0.055	0.050	0.059	0.050	0.413
4	4	0.3	0.052	0.050	0.054	0.050	0.273
4	4	0.5	0.052	0.049	0.053	0.049	0.180
4	4	0.7	0.052	0.050	0.053	0.050	0.142
4	4	0.9	0.051	0.050	0.052	0.050	0.117
10	3	0.1	0.053	0.050	0.054	0.050	0.357
10	3	0.3	0.052	0.050	0.052	0.050	0.204
10	3	0.5	0.052	0.050	0.051	0.050	0.138
10	3	0.7	0.051	0.050	0.051	0.050	0.106
10	3	0.9	0.050	0.049	0.051	0.049	0.090

**Table 5:** Frequency of Type I error at significance level 0.05 and frequency of a non-positive variance estimate, using the split-plot model (12).

*a*	*b*	*c*	$\sigma_{\text{AB}}^{2}$	R	SAS nobound	SAS default	Exact F-test	Pr(${\hat{\sigma }}_{\text{B}}^{2}\leq 0)$
2	4	12	0.1	0.013	0.050	0.055	0.050	0.257
2	4	12	0.3	0.033	0.051	0.059	0.051	0.099
2	4	12	0.5	0.040	0.050	0.058	0.050	0.055
2	4	12	0.7	0.043	0.050	0.057	0.050	0.040
2	4	12	0.9	0.047	0.050	0.057	0.050	0.029
3	2	12	0.1	0.000	0.042	0.089	0.051	0.329
3	2	12	0.3	0.006	0.044	0.102	0.050	0.188
3	2	12	0.5	0.014	0.046	0.092	0.050	0.123
3	2	12	0.7	0.022	0.048	0.086	0.050	0.093
3	2	12	0.9	0.028	0.048	0.080	0.049	0.076
3	4	12	0.1	0.030	0.050	0.054	0.050	0.139
3	4	12	0.3	0.046	0.050	0.052	0.050	0.025
3	4	12	0.5	0.050	0.050	0.052	0.050	0.008
3	4	12	0.7	0.051	0.051	0.053	0.051	0.004
3	4	12	0.9	0.051	0.050	0.053	0.050	0.002
3	4	5	0.1	0.018	0.050	0.046	0.049	0.287
3	4	5	0.3	0.036	0.050	0.054	0.049	0.108
3	4	5	0.5	0.043	0.050	0.054	0.051	0.048
3	4	5	0.7	0.047	0.050	0.054	0.050	0.028
3	4	5	0.9	0.049	0.050	0.054	0.050	0.020
4	4	12	0.1	0.039	0.050	0.053	0.051	0.087
4	4	12	0.3	0.050	0.049	0.050	0.050	0.009
4	4	12	0.5	0.050	0.050	0.051	0.050	0.001
4	4	12	0.7	0.050	0.050	0.052	0.050	0.000
4	4	12	0.9	0.051	0.050	0.051	0.050	0.000
10	3	12	0.1	0.047	0.050	0.052	0.050	0.021
10	3	12	0.3	0.051	0.051	0.051	0.050	0.000
10	3	12	0.5	0.050	0.051	0.051	0.051	0.000
10	3	12	0.7	0.051	0.050	0.051	0.050	0.000
10	3	12	0.9	0.050	0.050	0.051	0.050	0.000

Analyses were performed using the lmer and Anova functions of R and the mixed procedure of SAS. In R, type 2 tests were applied with contr.sum parametrization. Also in SAS, type 2 tests were used. The choice of type of test should not matter, as all designs were balanced. In SAS, the analyses were performed both with and without the nobound option. The Kenward and Roger method [18, 19] for computation of denominator degrees of freedom was applied as implemented in the software. In addition to these likelihood-based procedures, the null hypotheses were tested using the exact F-tests (11) and (14), utilizing the anova procedure of SAS for computation of sums of squares. For the exact F-tests, denominator degrees of freedom were calculated as (*a* − 1)(*b* − 1).

For the specific layout {*a* = 3, *b* = 2, *c* = 12} in model (12) with parameter values $\sigma_{e}^{2}=1$, $\sigma_{b}^{2}=1$ and $\sigma_{AB}^{2}=0.1$, an additional simulation study was performed, at which the null hypothesis *H*_0_ : *μ*_1._ = *μ*_2._ = … = *μ*_*a*._ was tested using type 3 tests. In R, using the lmer function, the contr.sum parametrization was applied. In SAS, using the mixed procedure, tests were made both with and without the nobound option. Furthermore, the exact F-test (14) was included. This simulation study comprised 10 000 simulated datasets. The quantiles of the obtained *p*-values were plotted against the quantiles of the continuous uniform distribution *U*(0, 1).

### Results

Table 4 shows the results of the simulation study using model (10). In R, with regard to all cases, frequency of Type I error was slightly too high. Especially when $\sigma_{B}^{2}$ was low, larger values than 0.05 were obtained, i.e., the null hypothesis was rejected somewhat too frequently. In those cases, the block variance, $\sigma_{B}^{2}$, was more often estimated as zero than in other cases. In SAS, frequency of Type I error was close to the nominal level 0.05 in all cases, provided the nobound option was applied, except for the layout {*a* = 3, *b* = 2}. However, using the default setting of the mixed procedure, i.e., without using the nobound option, frequency of Type I error was consistently too large. A positive correlation was observed between frequency of Type I error and frequency of block variance estimated as zero. As expected, the exact method showed frequencies of Type I error close to the nominal level.

Table 5 provides the results for model (12). In R, with layouts {*a* = 2, *b* = 4, *c* = 12} and {*a* = 3, *b* = 2, *c* = 12}, frequency of Type I error was much lower than the nominal level 0.05. In all examples, it was noticed that for lower values of $\sigma_{AB}^{2}$, frequency of Type I error was below 0.05. Using the mixed procedure with the nobound option, frequency of Type I error was close to the nominal level 0.05 in all cases, except with layout {*a* = 3, *b* = 2, *c* = 12}, where Type I error rates were too low. Using the mixed procedure with default settings, frequency of Type I error was almost always higher than the nominal level 0.05. Very high frequencies were observed for the problematic layout {*a* = 3, *b* = 2, *c* = 12}. It is noteworthy that results obtained with R and the default setting of SAS were sometimes very different. With the layout {*a* = 3, *b* = 2, *c* = 12}, frequency of Type I error varied between 0.00 and 0.03 in R, and between 0.08 and 0.10 in SAS. This layout was characterized by relatively large probabilities for a non-positive estimate of the main-plot variance $\sigma_{AB}^{2}$.

**Fig 1:**
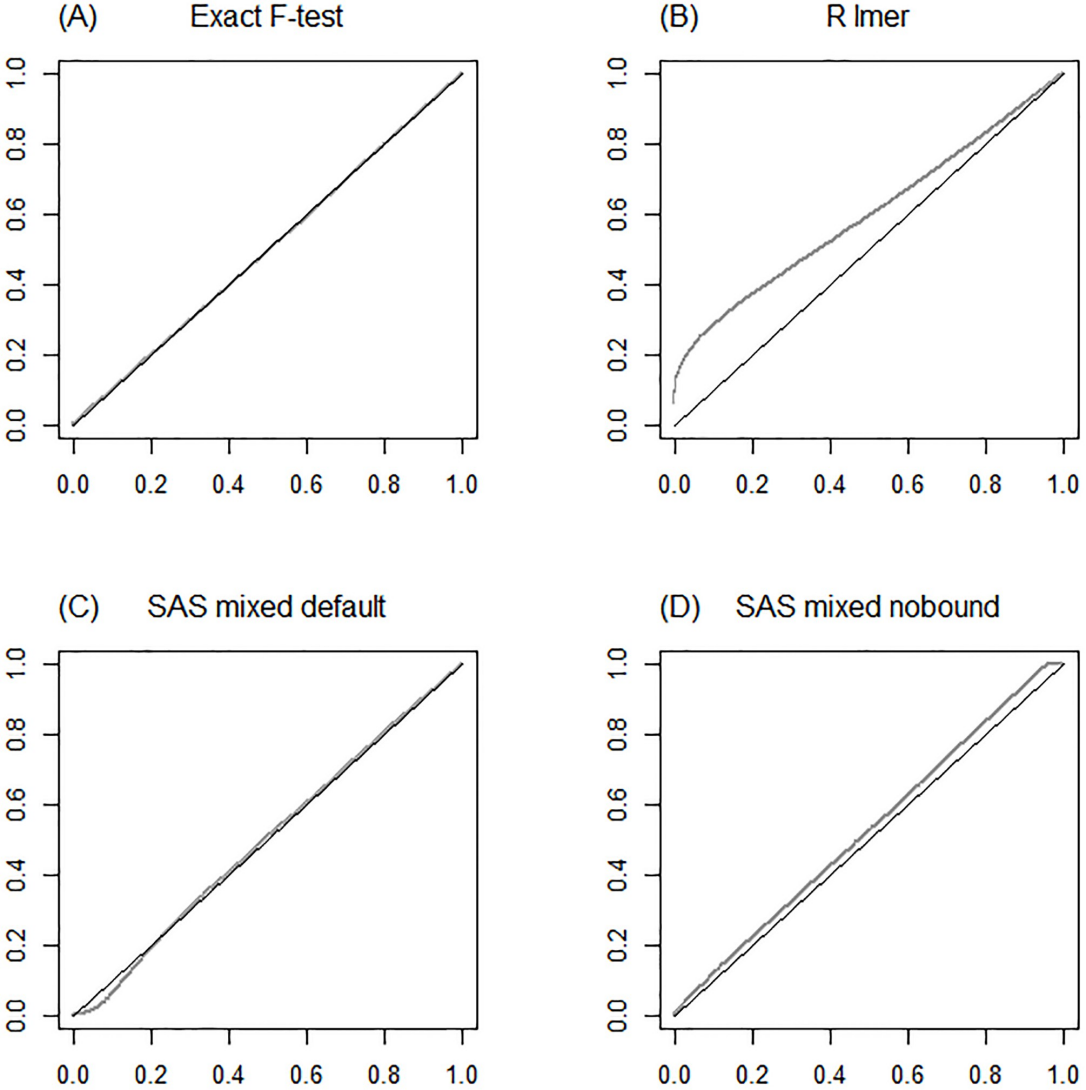
Quantiles of observed *p*-values, testing *H*_0_ : *μ*_1._ = *μ*_2._ = *μ*_3._ versus quantiles of the uniform distribution *U*(0,1). Based on 10 000 simulated datasets using model (12) with layout {*a* = 3, *b* = 2, *c* = 12} and parameter values $\sigma_{\text{e}}^{2}=1$, $\sigma_{\text{b}}^{2}=1$ and $\sigma_{\text{AB}}^{2}=0.1$. (A) Exact F-test. (B) R using the lmer and anova functions. (C) SAS using the mixed procedure with default settings. (D) SAS using the mixed procedure with the nobound option.

The results of the additional simulation study are presented in Fig 1. In these Q-Q plots, the performance of the methods can be studied for arbitrary levels of significance. As shown in Fig 1(A), the exact method (14) gave a uniform distribution of *p*-values, as expected. In Fig 1(B), the curve is above the reference line, indicating too many non-significant results when using the lmer function of R. With this method, the null hypothesis is rejected too rarely, due to the *F*-value being smaller than with the exact method (Table 3, Example 2).

The curve in Fig 1(C), for the mixed procedure of SAS using the default setting, is notably below the reference line at low levels of significance. Thus, when testing at significance levels 0.05 or 0.10, too many significant results are obtained using this method. This is a consequence of too many denominator degrees of freedom as compared to the exact method (Table 3, Example 2). For the mixed procedure using the nobound option, the curve of Fig 1(D) runs above the line. Although this is particularly so at large levels of significance, also at small levels e.g., 0.05, too few significant results are obtained (Table 5, design {*a* = 3, *b* = 2, *c* = 12}).

## Practical advice

This article discussed two issues: i) type 3 tests, and ii) non-positive estimates of variance components. The first issue is a problem, since the Anova and ezANOVA functions of R sometimes perform type 3 tests incorrectly. The second issue is a problem too, since the actual significance level, i.e., the true probability of incorrectly rejecting the null hypothesis, may differ from the nominal significance level. Here, we give some practical advice on how to work around these problems in R and SAS when possible.

Correct type 3 tests are obtained in R using the Anova function of the car package if the line

options(contrasts = c(''contr.sum'', ''contr.poly''))

is included in the beginning of the R script. This code changes the contrasts globally. The code

contrasts(A) <- contr.sum

is useful if the change is to be made only for factor A. Note that the Anova function is spelled with an initial capital A and is different from the anova function. Just like the Anova function, the ezANOVA function is dependent on the choice of contrast, so the default contr.treatment should not be used for type 3 tests, but contr.sum may be employed instead. Alternatively, the aov3 or the GLM function of the sasLM package may be preferred. These functions give correct type 3 tests even when contr.treatment is used. For mixed-effects models, correct type 3 tests are provided by the lmerTest package. These are obtained using the type argument of the anova function when applied to a model fitted by the lmer function. Note that the lmerTest package must be loaded before the model is fitted. No specific options are needed for correct type 3 tests using the glm and mixed procedures of SAS.

For mixed-effects models and balanced datasets, exact F-tests based on sums-of-squares computations, can be obtained using the test statement of the anova and glm procedures of SAS, where the user specifies the correct error terms. Similarly in R, exact F-tests can be computed using the aov function, which allows for a single error term in addition to the residual error term. For split-plot experiments, the sp.plot function of the agricolae package [34] is another option.

For analyses using the mixed procedure of SAS, we recommend allowing negative estimates of variance components when the main purpose is to draw conclusions about the fixed effects. This is made possible by the nobound option. Unfortunately, a similar option is not available for the lmer function. Although the tests are correctly implemented, the actual significance level deviates from the nominal one, as assessed in the Simulation section. Exceptional results such as discussed in the last paragraph of the Examples section might in R be avoided by using contr.sum.

## Discussion

Using different statistical software, a broad variation in results can be observed, even though the same statistical model is assumed. Hence, the accuracy of the results renders the choice of the statistical method for data analysis essential. This article investigated the glm procedure in SAS and several functions for analysis of variance in R, as applied to an unbalanced dataset with two factors in a crossed design. Furthermore, the mixed procedure of SAS and the lmer function of the lme4 package in R were studied with focus on balanced randomized complete block and split-plot experiments.

In unbalanced fixed-effects experiments, exact F-tests can be calculated from three different types of sums of squares, known as types 1, 2, and 3. While in balanced data, these are identical, in unbalanced data they correspond to different hypotheses [4, 9, 27]. The hypotheses of the type 3 tests do not depend on the number of observations in each treatment combination, and are therefore often preferred when analyzing unbalanced data. In addition to the choice of type of test, the user of R needs to select how the parameters of the factors should be constrained by coding of the factor levels, known as contrast functions. Here, three such contrasts were investigated; contr.treatment, contr.sum and contr.SAS [24]. Correct type 3 tests of no differences between the marginal means are obtained with the glm procedure of SAS and the sasLM package in R. Correct type 3 tests are also obtained with the Anova and ezANOVA functions of R when using contr.sum [35]. However, incorrect results are obtained in R with these functions when the default parametrization, contr.treatment, is applied. The tests obtained using this default contrast in R are not type 3 tests of marginal means, but tests of means conditioned on the first level of the other factor.

There is a misconception that type 3 tests are only correct if orthogonal coding is used [36]. According to the help page for the Anova function of the car package, type 3 tests ‘will normally only be sensible when using contrasts that, for different terms, are orthogonal in the row-basis of the model, such as those produced by contr.sum, contr.poly, or contr.helmert, but not by the default contr.treatment.’ This statement is misleading. The type 3 test is sensible regardless of the contrast used, provided it is correctly computed. The type 3 test is equivalent to Yates’s method of weighted squares of means [28], which does not result from zero-sum restrictions [4, 6]. In fact, the term ‘type 3 test’ origins from the SAS software [6], where orthogonal coding is not used [2]. In R terminology, the type 3 test is not dependent on which contrast is employed. However, the so called ‘type 3’ test provided by the Anova function of the car package is dependent on the choice of contrasts. In this article, we have explained what hypotheses the Anova function is actually testing.

In linear mixed-effects modelling of experiments, estimation of variance components and computation of denominator degrees of freedom, associated with inference on the fixed effects, are important parts of the analysis. Using the lmer function of R, variance estimates are always bounded at zero. Similarly, the mixed procedure of SAS has a default lower boundary constraint of zero. With variance components estimated to zero, the denominator degrees of freedom of the *F*-statistics are calculated differently in R and SAS, despite both software employing the Kenward and Roger method.

In the simulation study, Type I error rate was investigated, having the variance components either constrained at zero or not, using the different options offered by the software. With the default setting of SAS, higher frequencies of Type I error were observed in SAS than in R, due to the fact that the number of degrees of freedom is calculated differently and is higher in SAS. Thus, significant results are obtained more often with SAS than with R. Using the nobound option of the mixed procedure, i.e., allowing negative estimates of variance components, the frequency of Type I error was close to the correct value. This method can be recommended since it controls Type I error [37]. However, in our study, inaccurate results (*F* = 0) occasionally occurred when using the nobound option. For more complicated models than those investigated in this article, the default setting of the mixed procedure may be preferable for practical reasons, as problems with convergence may presumably arise more frequently with the nobound option.

Negative estimates of variance components, as allowed by the nobound option, may seem strange since variances cannot be negative. It is certainly challenging to explain the occurrence of negative estimates of variances. Furthermore, when any variance component is negative, it does not make sense to calculate the total variance and the components’ percentages of this total variance, as may otherwise be done. It is an old question how to act when estimates of variances become negative [3, 11]. One recurring suggestion is to replace negative estimates with zero, but such procedure disturbs the properties of the estimates. In particular, the distribution of the *F*-statistic is affected so that it is no longer F-distributed.

Linear mixed-effects models are very useful for analysis of complicated datasets, but hypothesis tests are usually approximate. For statistical analysis of simple experiments, there are exact methods based on sums of squares calculations, which should be preferred.

## Supporting information

S1 File R script for Table 2. (R)

S2 File SAS program for Table 2. (SAS)

S3 File The dataset for Example 1 of Table 3. (TXT)

S4 File The dataset for Example 2 of Table 3. (TXT)

S5 File The dataset for Example 3 of Table 3. (TXT)

S6 File R script for Example 1 of Table 3. (R)

S7 File R script for Example 2 of Table 3. (R)

S8 File R script for Example 3 of Table 3. (R)

S9 File SAS program for Example 1 of Table 3. (SAS)

S10 File SAS program for Example 2 of Table 3. (SAS)

S11 File SAS program for Example 3 of Table 3. (SAS)
