# Correction: Notes on correctness of *p*-values when analyzing experiments using SAS and R

**DOI:** 10.1371/journal.pone.0296235

**Published:** 2023-12-18

**Authors:** The PLOS ONE Staff

There is an error in Table 2. In the Methods column, ‘R proc glm’ should be ‘SAS proc glm’. The publisher apologizes for this error. Please see the correct Table 2 here.

**Table 2:** Results from analyses of the unbalanced two-factorial experiment using the lm, anova and Anova functions of R and the glm procedure of SAS: *F-*statistics and *p-*values for the test of sex.

Method	Type 1		Type 2		Type 3	
	*F*	*p*	*F*	*p*	*F*	*p*
R contr.treatment	10.9622	0.0091	3.8073	0.0828	5.9595	0.0373
R contr.sum	10.9622	0.0091	3.8073	0.0828	0.5151	0.4911
R contr.SAS	10.9622	0.0091	3.8073	0.0828	13.6824	0.0049
SAS proc glm	10.9622	0.0091	3.8073	0.0828	0.5151	0.4911
